# The Implementation Science for Genomic Health Translation (INSIGHT) Study in Epilepsy: Protocol for a Learning Health Care System

**DOI:** 10.2196/25576

**Published:** 2021-03-26

**Authors:** Elena Valeryevna Feofanova, Guo-Qiang Zhang, Samden Lhatoo, Ginger A Metcalf, Eric Boerwinkle, Eric Venner

**Affiliations:** 1 Human Genetics Center Department of Epidemiology, Human Genetics and Environmental Sciences, School of Public Health The University of Texas Health Science Center at Houston Houston, TX United States; 2 Department of Neurology The University of Texas Health Science Center at Houston Houston, TX United States; 3 School of Biomedical Informatics The University of Texas Health Science Center at Houston Houston, TX United States; 4 Texas Institute for Restorative Neurotechnologies The University of Texas Health Science Center at Houston Houston, TX United States; 5 Human Genome Sequencing Center Baylor College of Medicine Houston, TX United States

**Keywords:** genomic medicine, electronic health record, implementation, genetics, prototype, decision support

## Abstract

**Background:**

Genomic medicine is poised to improve care for common complex diseases such as epilepsy, but additional clinical informatics and implementation science research is needed for it to become a part of the standard of care. Epilepsy is an exemplary complex neurological disorder for which DNA diagnostics have shown to be advantageous for patient care.

**Objective:**

We designed the Implementation Science for Genomic Health Translation (INSIGHT) study to leverage the fact that both the clinic and testing laboratory control the development and customization of their respective electronic health records and clinical reporting platforms. Through INSIGHT, we can rapidly prototype and benchmark novel approaches to incorporating clinical genomics into patient care. Of particular interest are clinical decision support tools that take advantage of domain knowledge from clinical genomics and can be rapidly adjusted based on feedback from clinicians.

**Methods:**

Building on previously developed evidence and infrastructure components, our model includes the following: establishment of an intervention-ready genomic knowledge base for patient care, creation of a health informatics platform and linking it to a clinical genomics reporting system, and scaling and evaluation of INSIGHT following established implementation science principles.

**Results:**

INSIGHT was approved by the Institutional Review Board at the University of Texas Health Science Center at Houston on May 15, 2020, and is designed as a 2-year proof-of-concept study beginning in December 2021. By design, 120 patients from the Texas Comprehensive Epilepsy Program are to be enrolled to test the INSIGHT workflow. Initial results are expected in the first half of 2023.

**Conclusions:**

INSIGHT’s domain-specific, practical but generalizable approach may help catalyze a pathway to accelerate translation of genomic knowledge into impactful interventions in patient care.

**International Registered Report Identifier (IRRID):**

PRR1-10.2196/25576

## Introduction

Despite enormous progress in gene discovery and the prioritization of research objectives related to precision medicine, translation of this rich body of scientific knowledge into standard patient care has been slow. Clinical genomics is emerging as a standard of care, with more than 60% of US private payers covering multigene testing in cancer [1] and in congenital anomalies and neurodevelopmental disorders in children [2]. Whole exome sequencing (WES) produces a considerable diagnostic yield (DY) and results in meaningful clinical management changes in a number of heritable diseases, including cardiomyopathies (DY=12%-50%) [3-5], chronic kidney disease (DY=10%-24%) [6-8], neuromuscular diseases (DY=39%) [9], hearing impairment (DY=33.5%) [10], primary immunodeficiency (DY=56%) [11], hematological disorders (DY=17%-23% for bleeding diathesis and pediatric platelet disorders) [12,13], monogenic conditions (DY=25%-58%) [14], and lung and colorectal adenocarcinomas (DY for the germ-line mutations ~5%; DY for somatic mutations ~30%) [15]. In epilepsy, WES provides a DY of approximately 45% [16-18], with approximately 40% of these having potential treatment implications [19,20].

Overall, the number of genetic studies, with ever-increasing sample sizes and discovery rates, have been growing [21]. At the same time, it has been estimated that 3% or less of the published research is focused on development and integration of evidence-based guidelines into clinical practice [22], with an average of 17 years passing between obtaining scientific evidence and its integration into clinical care [23]. Further, broad implementation of genomics information into standard clinical practice faces a significant gap in management velocity, defined as the time taken for new information to initiate change or to advance diagnosis and/or treatment.

In the area of genomic medicine, we are addressing the management velocity gap by building software and workflows between point of clinical care, the sequencing laboratory, variant interpretation, electronic health records (EHRs), and clinical decision support, thus streamlining the process of ordering, sample collection, sequencing, interpretation, and return of results. The benefit is identification of clear care pathways for placing testing into routine health care, with implications generalizable to other complex diseases. We designed the communication pathways to ensure that clinicians have access to interpreted genetic data and that rich phenotyping is available for researchers to mine for new diagnostic and prognostic discoveries. In particular, mining phenotype data from EHRs has been shown to be especially useful [24].

Several features of epilepsy set epilepsy care apart from many other conditions, making it an ideal setting in which to develop novel user-oriented EHR systems. This complex chronic neurological disease affects 50 to 60 million people worldwide and 3 million lives in the United States [25,26]. The high prevalence of epilepsy is in contrast to lower prevalences of neuromuscular diseases (1 to 10 per 100,000 people) [27], hypertrophic (estimated 98,958 cases in the United States) and dilated (1 in 250 to 1 in 500 adults) cardiomyopathies [28,29], all lung cancer cases (n=571,340), and all colon and rectum cancer cases (n=1,544,570) [30]. Only hearing loss has a higher prevalence of approximately 60.7 million people in the United States [31], but cannot compare in the severity of the loss of quality of life. Epilepsy affects people of all ages, with bimodal age incidence peaks in early life and in the elderly and the greatest prevalence in adult and young adult populations [25,32]. Thus, it is an excellent model for clinical genomics knowledge integration spanning pediatric, adult, and geriatric clinical care settings. In contrast, colon and lung cancer, chronic kidney diseases, and cardiomyopathies are predominantly seen in older adults [29,30]. Half of all patients with epilepsy have medical, psychiatric, and/or cognitive comorbidities [33,34], requiring evidence-based management interactions as genomic knowledge accrues. Multiple treatment approaches are available for epilepsy management, ranging from ketogenic diets to surgery, in contrast to neuromuscular diseases, which are predominantly managed by immunotherapy [35], and cancer, which usually requires a surgical intervention [36].

Around one-third of epilepsy patients are unresponsive to antiepileptic drugs (AEDs) [37], similar to other heritable drug-resistant conditions (eg, early-onset inflammatory bowel diseases) [38,39]. Adverse drug reactions (ADRs) are seen in 6% to 7% of all hospital admissions, include life-threatening reactions, and remain a vexing issue in epilepsy management [40]. One-third of all AEDs have known pharmacogenomics (PGx) loci [41,42]. Information on a patient’s genetic status for these loci can predict occurrence of ADRs and affect a clinician’s management strategy [41,42]. Epilepsy can model the adaptation of actionable PGx into clinical settings for other diseases, with lessons learned impacting the data presentation, decision support, and reanalysis as guidelines are updated. Moreover, there are many heterogeneous subtypes of epilepsy that are difficult or impossible to distinguish without molecular data. Genomic data can aid in diagnosis, but are also prone to discovery of variants of unknown significance (VUS) [43]. In turn, EHR data contain a rich phenotype allowing for improved variant interpretation [44], which can help reduce VUS. 

However, traditional EHR systems, such as Epic and Cerner, do not allow for genomic information to be entered in a structured, action-ready format [45]; such integration involves expensive customization and workflow reconfiguration efforts and costs, representing fundamental barriers [45]. As such, systems were not designed to take advantage of the latest genomic medicine advances, nor are they amenable to prototyping multiple approaches to optimize the presentation and collection of data. Hence, there is a need for exemplary user-oriented systems built to prototype and evaluate EHR functionality. In this project, we seize the unique opportunity presented by our control over the development of EHR and clinical reporting environments to integrate genomic knowledge with phenotypic information and evaluate its clinical utility, and propose an adaptable clinical genomics workflow design.

## Methods

### Development of Health Data Management Platforms

To bring together the epilepsy clinic and clinical genomics information, a connection needs to be established between respective health data management platforms. Previously, we developed the Epilepsy Tracking and optimized Management engine (EpiToMe), an EHR system customized for epilepsy care, to address common EHR challenges, such as inability to handle specialty-specific documentation requirements [46]. EpiToMe uses agile, physician-centered development to optimize clinical workflow and ease patient care documentation and billing [46]. EpiToMe captures data in clinical research–ready form and links it with hospital EHR systems, while providing integrated interfaces for patient tracking, report generation, structured data capture for electroencephalogram (EEG) reporting, daily video-EEG reporting, and comprehensive presurgical phase reporting for Epilepsy Monitoring Units (EMUs). This is achieved using a metadata-anchored approach: we used the Epilepsy and Seizure Ontology as the semantic anchor for implementing all front-end and back-end capabilities of EpiToMe. Operationalization of EpiToMe at the University of Texas Health Science Center at Houston (UTHealth) (an Epic site) and Memorial Hermann Health System (a Cerner site) since February 2019 has demonstrated its effectiveness to support clinical workflows, with 21,456 EEG reports, 4534 EMU daily reports, and 2635 EMU phase reports completed.

The Human Genome Sequencing Center’s (HGSC) clinical reporting platform, Neptune, enables identification and reporting of known disease-causing variants in gene sets of interest, the curation of potential novel pathogenic variants, and sharing of important—or VIP—variants with clinical partners. Neptune searches for each sample’s genomic variants in a curated database, currently containing 381,564 variants annotated with a wide array of information drawn from public resources (ie, ClinVar, Online Mendelian Inheritance in Man [OMIM], and literature review) and internal curation data sets. If all variants are properly curated, Neptune allows for automated reporting and produces a clinical report containing pathogenic single-nucleotide variants and copy number variants, other project-specific report elements (eg, PGx output and polygenic risk scores), descriptive text, coverage statistics produced by the HGSC’s Exome Coverage and Identification Report software, and other required reporting elements (eg, sample metadata and methodology) for review and approval by a laboratory director. Neptune has been applied to Electronic Medical Records and Genomics (eMERGE) III and HeartCare samples, with more than 15,000 clinical reports generated to date [47,48]. Neptune supports a variety of output formats, including human-readable HTML and PDF as well as structured JavaScript Object Notation (JSON), XML, and Fast Healthcare Interoperability Resources (FHIR) formats. The eMERGE III project addressed a similar challenge: to create a functioning network of existing clinical laboratory reporting systems and clinical site EHRs [48]. Key lessons from this project were the importance of using open interoperability standards, of designing around regular updates to genomic data, and of structured genomic data for clinical decision support.

Last, the importance of genetic information in routine cardiovascular health management has been demonstrated through our earlier HeartCare gene panel. Pilot-testing in 700 participants resulted in identification of pathogenic and likely pathogenic variants (8%), identification of PGx loci (51%) [47], and prompting clinical management changes, including medication and imaging (6%), referral (15%), and additional laboratory testing (79%).

### Integrated Clinical Genomics Platform

An adaptable clinical genomics workflow requires formation of an integrated clinical genomics platform, which consists of two stages, as described below.

#### Intervention-Ready Genomic Knowledgebase

Epilepsy patients are routinely assessed with magnetic resonance imaging, blood tests, EEG, and, occasionally, urine tests and skin biopsies. Additionally, AED prescriptions entail monitoring of AED and sodium levels, blood counts, and liver and bone function [49-52]. These tests provide data essential for an initial diagnosis and reside in the EHR. EpiToMe schema can be extended to store existing phenotype data alongside genomic information, thus linking patient records with gene and variant-level annotations (eg, inheritance pattern, gene-disease association, drug-gene PGx pairs, mutational effect, variant interpretation, associated evidence codes, and literature; see Multimedia Appendices 1 and 2). Since genomic annotations change often, our model includes keeping annotations up to date and providing reports and alerts of important changes. This involves identification and creation of mappings for test results to Human Phenotype Ontology (HPO) terms [53,54], with automatic transfer to Neptune. Existing HPO-gene mappings can be employed for genes prioritization [55]. Subsequently, this resource can be used to develop electronic phenotyping to detect patients who are likely to benefit most from genetic analysis. 

#### Health Informatics Platform and Systems Coupling

We designed a health informatics platform called EpiToMe+ that brings the Intervention-Ready Genomic Knowledgebase (iRGK) to bear with clinical decision support and patient care. To achieve a genomics-integrated epilepsy care system, EpiToMe+ is designed to extend capabilities as follows:

1. Expand clinical report interfaces with a genomic variants report [46], which is designed to reflect the status and contents of biospecimen obtainment and genetic report steps of the clinical genomics workflow.
2. Add a genetic testing step to the tracker—an interactive, real-time interface—displaying patient status in the epilepsy care workflow [46].
3. Integrate iRGK for actionable PGx loci into EpiToMe’s clinical report [46], with built-in notification when an AED with a known actionable PGx locus present in the patient is prescribed, to help improve patient management.
4. Clinical laboratory information, prescribed medications, exam data, patient history, and genetic information are then transmitted from Cerner and Epic systems, using Health Level Seven International (HL7) messaging, and are used for ADR prediction and identification [56,57].
5. Trained statistical models for ADR prediction on available EpiToMe data can then be incorporated to produce a summary of the estimated risks of ADRs, as well as existing risk factors, to support clinical decision making [58,59].

Integration between Neptune and EpiToMe+ enables genomic data to be acted upon at the point of care.

### Clinical Genomics Workflow

**Figure 1:**
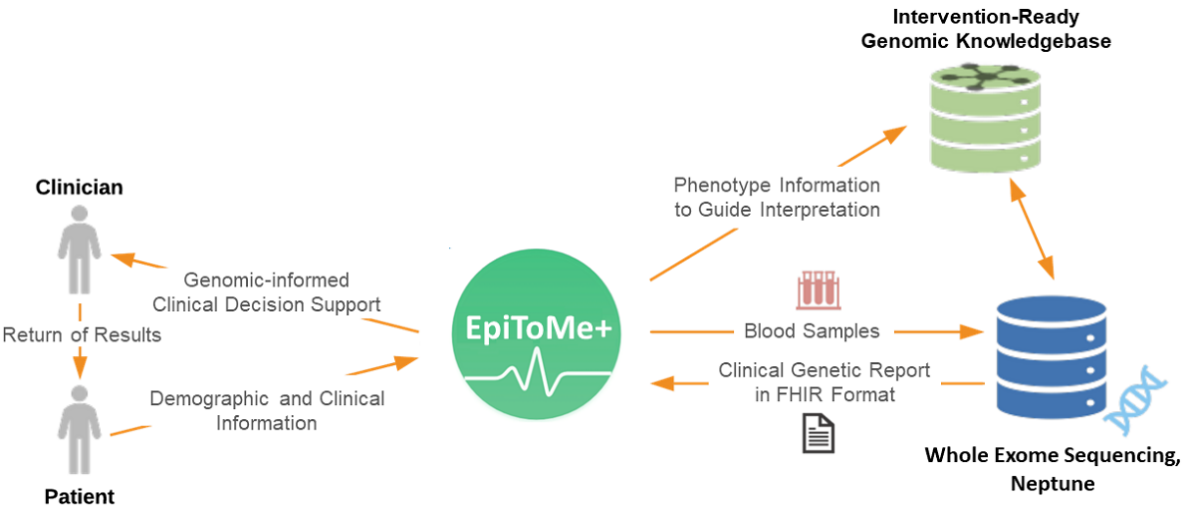
Clinical genomics workflow. The Implementation Science for Genomic Health Translation (INSIGHT) project has been designed to connect two existing clinical informatics systems: Epilepsy Tracking and optimized Management engine (EpiToMe), a bespoke epilepsy-specific electronic health record system, and Neptune, a clinical genomics reporting pipeline. To pilot this system, 120 patients from the Texas Comprehensive Epilepsy Program for whole exome sequencing are to be recruited. Their genetic data are designed to be generated and analyzed at the Human Genome Sequencing Center and returned via Fast Healthcare Interoperability Resources (FHIR) messages to EpiToMe+, where it can be presented at the point of care, with clinical decision support. A new knowledge base, Intervention-Ready Genomic Knowledgebase, is designed to form the back end, enabling this integration.

Our overall clinical workflow consists of five steps (see Figure 1).

#### Demographic and Clinical Information

During a patient visit, demographic and clinical information is created and updated through EpiToMe’s existing HL7 messaging with a parent EHR. Clinical reports interface into EpiToMe, capturing patient information, including EEG reporting, phase reporting, daily care reporting, and evoked potentials [46]. HL7 is a widely used framework and interoperability standard for clinical and administrative data transmission between EHR systems [60]. HL7 engine design ensures seamless transfer of obtained data between EpiToMe and the parent EHR [46].

#### Blood Samples

Blood is drawn following consent and sent to the HGSC Clinical Laboratory. DNA extraction and biobanking is performed during this step.

#### Sequencing, Genomic Analyses, and Interpretation

Sequencing, genomic analyses, and interpretation are performed at the HGSC with results stored in Neptune. Sample preparation and sequencing are followed by mapping, alignment, and variant calling using standard clinical pipelines. Neptune selects novel variants according to reporting requirements for review according to American College of Medical Genetics (ACMG) guidelines [61]. For each sample, a deidentified clinical report is developed to contain clinically relevant variants and PGx loci for AEDs to help improve patient management. Pharmacogenomic alleles are detected by force-calling genotypes according to their definitions in the Clinical Genetics Implementation Consortium (CPIC) and/or the Pharmacogenomics Knowledge Base (PharmGKB). For the challenging cytochrome P450 2D6 (Cyp2D6) region, we anticipate validating the DRAGEN Cyp2D6 caller [62]. Our reports are designed to focus on gene-drug pairs with CPIC evidence levels A and B and/or PharmGKB levels 1A to 2B, especially those with existing therapeutic management recommendations according to the US Food and Drug Administration (see Multimedia Appendix 2) [41,42,63]. After review and approval by an ACMG-boarded lab director, the report is then transferred in a structured format to UTHealth for final report rendering and storage in the iRGK. Last, a local disease-specific reference library is designed to reflect current knowledge in the field to inform genetic analyses and clinical decision support.

#### Clinical Genetic Report

Reports of relevant genomic variants are sent from Neptune to EpiToMe+ using the HL7 FHIR genomic format. The previously developed HL7 FHIR is a rising standard for clinical genomics reporting [45,60,64], which can be adopted for structuring the required genomic data (ie, single-nucleotide variants, indels, copy number variants, and PGx), with revisions to the HL7 Clinical Genomics Work Group to be recommended as necessary. An FHIR-based interface enables reports of genomic variants transmitted to EpiToMe+ for automatic storage and reflection in genomic reports for individual patients. We designed system-to-system coupling to connect genomic reports with other clinical information so that the patient’s clinical information can be used to inform the interpretation of genetic results and to capture phenome-genome interactions within the EHR data. For example, the use of existing biochemical data can lead the reviewer to specific gene pathways, which, in turn, may help with disease stratification. This step also addresses the genetic information storage issue, since traditional EHR systems are not prepared to accommodate the complete genetic results, which tend to be bulky [45]. The EpiToMe architecture ensures effective and secure storage of patients’ data.

#### Clinical Decisions and Return of Results

The resulting clinical decisions and care are provided by epilepsy physicians trained to effectuate available clinical genomic knowledge. Return of clinical genomics results (ie, return of results [RoR]) for common chronic diseases is challenging. We found previously that for positive findings, clinicians prefer to perform RoR personally, due to their close relationships with the patient (manuscript in preparation). Further, 40% of epilepsy patients have psychiatric comorbidities [65], and clinicians are trained to handle complex clinical care scenarios. In addition, physicians are generally not sufficiently prepared to integrate genetic test results into clinical care, with continuing professional development being particularly important [66]. Genetic counselors, although extensively trained for Mendelian disorders, birth defects, inherited cancer, and inborn errors of metabolism, may lag behind the expanding knowledge in other areas, and collaborations between genetic counselors and clinicians can ensure adequate interpretation of genetic information [67].

In our model, negative findings are returned in a form letter, positive findings are returned by the patient’s clinician, and in cases of complex genetic disease or high heritable risk, patients are referred for genetic counseling. Our model also includes “scripts” that guide clinicians through common findings. For instance, for carriers of the G allele of rs121964976 (*GLDC*, ClinVar No. VCV000011985), recommendations can include a ketogenic diet, protein restriction, or sodium benzoate [68,69], while patients carrying rare pathogenic *POLG* alleles, such as rs113994098 (ClinVar No. VCV000013502.10), should not be prescribed valproate for seizure control (see Multimedia Appendices 1 and 2) [63,70]. Further, the effectiveness with which clinicians return genetics test results can be measured by assessing, for example, the level of comprehension following RoR.

Compared to other existing clinical genetics workflows, such as those developed for HeartCare, All of Us, eMERGE, and rapid newborn intensive care unit projects, Implementation Science for Genomic Health Translation (INSIGHT) stands out due to the high positive rate in epilepsy and availability of detailed EHR phenotyping (see Table 1). It follows the best practices for the RoR and report contents. The usage of WES in INSIGHT is designed to allow for genetic data reanalysis for epilepsy, as well as for other diseases, and harbors a possibility for future research, extending beyond the preselected genetic loci in panel-based projects. The challenges, including phenotype collection and RoR approach, are shared across disease areas and projects. The multimodality aspect of epilepsy makes INSIGHT generalizable to other disease areas, further strengthening the case for INSIGHT as an exemplary clinical genetics workflow.

**Table 1:** Comparison of the main characteristics of selected clinical genetics workflows.

Workflow characteristic		HeartCare	All of Us	eMERGE^a^	INSIGHT^b^	Rapid NICU^c^
Genetic test type		Panel	Panel	Panel	WES^d^	WGS^e^
Approximate positive rate, %		8	~2-3	3	>40	>30
Up-front phenotype term collection		Main disease areas	None	Main disease areas	Detailed	—^f^
**Return of results**						
	Return of results by clinician	Yes	No	No	Yes	Yes
	Return of results by genetic counselor	Partial	Yes	Yes	Partial	Yes
**Report characteristics**						
	Form of report is easily EHR^g^-integratable	Yes	Somewhat	Yes	Yes	Yes
	Report is focused on one disease area	Yes	No	No	Yes	No
	Report contains pharmacogenomics	Yes	Yes	Yes	Yes	Yes
	Report contains polygenic risk score	Yes	No	No	No	No
**Usage of genetic information**						
	Reanalysis of genetic information desired	Yes	Yes	Yes	Yes	Sometimes
	Reanalysis enables future diagnosis on other diseases	No	Yes	Yes	Yes	Yes
	Supports genotype-phenotype analysis	Yes	Yes	Yes	Yes	Yes
**Generalizability**						
	Overall reporting framework generalizable to other diseases	Yes	Yes	Yes	Yes	Yes
	Generalization *requires* development of specialty-specific systems	No	No	No	No	No

^a^eMERGE: Electronic Medical Records and Genomics.

^b^INSIGHT: Implementation Science for Genomic Health Translation.

^c^NICU: neonatal intensive care unit.

^d^WES: whole exome sequencing.

^e^WGS: whole genome sequencing.

^f^Project is in development and data are not available.

^g^EHR: electronic health record.

## Results

INSIGHT was approved by the Institutional Review Board of UTHealth on May 15, 2020. It is designed as a 2-year study, set to start in December 2021. Activities of the first year consist of system testing, integration, and iRGK development. Activities of the second year include enrollment of 120 patients from the Texas Comprehensive Epilepsy Program to pilot-test the INSIGHT clinical genomics workflow, exercise communication between Neptune and EpiToMe+, identify integration bottlenecks, and validate the EpiToMe+ genomic reporting and clinical decision support interfaces. Following the principles of implementation science, this pilot is designed to undergo an evaluation and modification period prior to further scaling of the INSIGHT program. EpiToMe+ is designed to continually trace patient-level outcomes (seizure control, adverse event incidence, etc), to measure value of implementation (eg, genetic results turnaround time, clinician interactions with decision support tools, and response to PGx prescription notifications), and to guide improvement in quality and effectiveness of use of clinical genetics health services. The results of the study are expected in the first half of 2023.

## Discussion

We present a workflow and software, named INSIGHT, for integrating genomics into routine clinical care. INSIGHT is designed to allow evaluation of model data structures and infrastructure frameworks for an integrated clinical genomics platform, allowing for genetic testing, data incorporation, results interpretation, and clinical decision support, which can be universally applied. Epilepsy exemplifies various modalities of care and is a suitable exemplar to demonstrate innovation, feasibility, and potential clinical impact for patient care. Therefore, lessons learned will be applicable to a number of conditions in which clinical genomics already show promise (eg, cardiomyopathies, certain kidney and digestive disorders, and a list of familial or early-onset cancers). Additionally, our design includes assessment of the feasibility of adopting and extending existing data standards to integrate disparate data types into a single knowledge base. INSIGHT is designed to help catalyze a pathway to accelerate translation of genomic knowledge into impactful clinical intervention and patient care practice and to promote discovery.

It is, nonetheless, important to consider limitations of INSIGHT. First, some of its aspects are specific to epilepsy, which might raise questions about the generalizability of the tools. It is important to note that INSIGHT is generalizable and adaptable to other disease specialties in the following key aspects:

1. The conceptual architecture identifies and connects previously independent system components in disparate domains through a synergistic flow of data and information that can help drive the velocity of management.
2. The system interconnect leverages standard messaging formats, such as FHIR and HL7.
3. EpiToMe+ contains generalizable and reusable software components and interface innovations.
4. Neptune is an open source software [71] and has been applied to clinical reporting for a wide variety of disease contexts, including cardiac disease (ie, dyslipidemias, arrhythmias, and cardiomyopathies), breast and colon cancers, as well as samples with no indication for testing.

Second, creation of a VIP database for any disease area from the ground up requires a significant investment of time and resources. For the epilepsy-related genes, our primary focus will be on the potentially actionable genes, especially those having been reviewed by the ClinGen Epilepsy Gene Curation Expert Panel. By design, we will take into consideration the ACMG pathogenic and likely pathogenic variants reported to ClinVar. Hence, the VIP database for epilepsy is designed to be built on the available evidence and previous work and will be evolving over time.

The timeline for recruitment, data collection, sequencing, and RoR for 120 patients is designed to be relatively short. Fortunately, the epilepsy clinic of the Texas Comprehensive Epilepsy Program sees more than 600 new patients per year referred from the Greater Houston Metropolitan Area and elsewhere. Currently, less than 10% of patients undergo genetic testing. It is the goal of INSIGHT to follow an implementation science framework to scale genetic testing to be part of routine care. INSIGHT is designed to focus on intractability and focal or generalized epilepsy severity, defined by the presence of convulsive seizures at least once a month, since it is this group that is likely to benefit the most from genetic analysis and targeted treatments that promise greater efficacy as well as obviate ADRs. Besides, sequencing coverage is always a concern for tests based on next-generation sequencing. We have identified 1909 genes related to epilepsy and assessed their coverage in our Clinical Laboratory Improvement Amendments–validated exome assay. A total of 96% of coding regions defined by these genes have at least 20 times coverage, and 98.5% receive at least 10 times coverage.

In conclusion, there is a need for exemplary user-oriented systems built to prototype and evaluate EHR functionality in the genomics context. The development cycles for EHR systems are long, so prototyping new feature sets and evaluating them in a clinical context is an important avenue for speeding up the deployment of genomic medicine. The resource described here, embedded with the adaptable clinical workflow design, is designed to place a body of translational knowledge in the context of precision intervention in patient care, allowing us to benchmark and optimize its delivery.

## Appendix

Multimedia Appendix 1 Genes known to cause epilepsy and the corresponding existing and emerging therapeutic approaches.

Multimedia Appendix 2 Known pharmacogenomics loci in epilepsy.

## Abbreviations

ACMG: American College of Medical Genetics
ADR: adverse drug reaction
AED: antiepileptic drug
CPIC: Clinical Genetics Implementation Consortium
Cyp2D6: cytochrome P450 2D6
DY: diagnostic yield
EEG: electroencephalogram
EHR: electronic health record
eMERGE: Electronic Medical Records and Genomics
EMU: Epilepsy Monitoring Unit
EpiToMe: Epilepsy Tracking and optimized Management engine
FHIR: Fast Healthcare Interoperability Resources
HGSC: Human Genome Sequencing Center
HL7: Health Level Seven International
HPO: Human Phenotype Ontology
INSIGHT: Implementation Science for Genomic Health Translation
iRGK: Intervention-Ready Genomic Knowledgebase
JSON: JavaScript Object Notation
OMIM: Online Mendelian Inheritance in Man
PGx: pharmacogenomics
PharmGKB: Pharmacogenomics Knowledge Base
RoR: return of results
UTHealth: the University of Texas Health Science Center at Houston
VUS: variants of unknown significance
WES: whole exome sequencing
