# Supplementary material for: The Implementation Science for Genomic Health Translation (INSIGHT) Study in Epilepsy: Protocol for a Learning Health Care System
Source: JMIR Res Protoc. 2021 Mar 26;10(3):e25576. doi: 10.2196/25576 (PMC8088873; doi:10.2196/25576)
Supplement: Multimedia Appendix 1 [file resprot_v10i3e25576_app1.pdf]

Multimedia Appendix 1. Genes known to cause epilepsy and the corresponding existing and emerging therapeutic approaches.

Gene	OMIM Phenotype	Management
ADSL	Adenylosuccinase deficiency (OMIM:103050)	D-ribose and uridine administration; S-adenosyl-l methionine; ketogenic diet (limited evidence) [1].
ALDH7A1	Pyridoxine-dependent epilepsy (OMIM:266100)	Current treatment is pyridoxine or pyridoxal 5'-phosphate supplementation; lysine restricted diet; arginine supplementation [2, 3].
ALG13	Epileptic encephalopathy, early infantile, 36 (OMIM:300884); Congenital disorder of glycosylation, type Is (OMIM:300884)	First-line treatment - corticosteroids. Benzodiazepines - need further investigation. Ketogenic diet may be helpful [4].
AMT	Glycine encephalopathy (OMIM:605899)	Decrease glycine concentrations by sodium benzoate and low protein diet; N-methyl-D-aspartate receptor antagonist (i.e. dextromethorphan); anti-epileptic drugs [5, 6].
ATP7A	Menkes disease (OMIM:309400)	Injections with copper histidine, starting in neonatal period [7].
ATP1A3	CAPOS syndrome, (OMIM:601338); Alternating hemiplegia of childhood 2 (OMIM:614820); Dystonia-12 (OMIM:128235)	Most frequently used - levetiracetam, phenobarbital, valproic acid and topiramate [8]. Flunarizine [9]. Ketogenic diet [10].
BTD	Biotinidase deficiency, 3 (OMIM:253260)	Biotin therapy [11].
CACNA1A	Spinocerebellar ataxia 6 (OMIM:183086); Epileptic encephalopathy, early infantile, 42 (OMIM:617106); Migraine, familial hemiplegic, 1, with progressive cerebellar ataxia (OMIM:141500); Episodic ataxia, type 2 (OMIM:108500); Migraine, familial hemiplegic, 1 (OMIM:141500)	Lamotrigine (limited evidence) [12].
CAD	Epileptic encephalopathy, early infantile, 50 (OMIM:616457)	Uridine [13].
CDKL5	Epileptic encephalopathy, early infantile, 2 (OMIM:300672)	Consider vagus nerve stimulation [14]. Vigabatrin and zonisamide combination may be effective [15]. Conflicting data for ketogenic diet [16, 17].
CHRNA4	Nicotine addiction, susceptibility to (OMIM:188890); Epilepsy, nocturnal frontal lobe, 1 (OMIM:600513)	Transdermal nicotine showed promise in several patients [18].
DEPDC5	Familial focal epilepsy, with variable foci 1 (OMIM:604364)	DEPDC5 participates in inhibition of mTORC1 recruitment to the lysosomal membrane during nutrient deprivation, thereby preventing its activation. In mice, mTORC1 inhibition with rapamycin was effective in preventing seizures [19].

Gene	OMIM Phenotype	Management
EEF1A2	Epileptic encephalopathy, early infantile, 33 (OMIM:616409); Mental retardation, autosomal dominant 38 (OMIM:616393)	Tetrabenazine for hyperkinetic movements (limited evidence) [20].
FGF12	Epileptic encephalopathy, early infantile, 47 (OMIM:617166)	Sodium channel blockers [21].
FOLR1	Neurodegeneration due to cerebral folate transport deficiency (OMIM:613068)	Oral or intravenous folinic acid (5-formyltetrahydrofolate) supplementation [22].
GABRA1	Epilepsy, childhood absence, susceptibility to, 4 (OMIM:611136); Epilepsy, juvenile myoclonic, susceptibility to, 5 (OMIM:611136); Epileptic encephalopathy, early infantile, 19 (OMIM:615744)	Verapamil (limited evidence) [23]. Rs2279020 is associated with decreased risk of valprioc acid resistant epilepsy [24].
GABRB3	Epilepsy, childhood absence, susceptibility to, 5 (OMIM:612269); Epileptic encephalopathy, early infantile, 43 (OMIM:617113)	Clonazepam (limited evidence) [25].
GAMT	Cerebral creatine deficiency syndrome 2 (OMIM:612736)	Oral creatine monohydrate supplementation; L-ornithine supplementation; dietary arginine restriction [26]. Other possible approaches include dietary protein restriction and sodium benzoate and S-adenosylmethionine (SAM) in patients with genetic defects of SAM binding site of GAMT [26].
GLDC	Glycine encephalopathy (OMIM:605899)	Decrease glycine concentrations by sodium benzoate and low protein diet; N-methyl-D-aspartate receptor antagonisit (i.e. dextromethorphan); anti-epileptic drugs [5, 6, 27].
GNAO1	Epileptic encephalopathy, early infantile, 17 (OMIM:615473); Neurodevelopmental disorder with involuntary movements (OMIM:617493)	Tetrabenazine and deep brain stimulation (limited evidence) [28].
GOSR2	Epilepsy, progressive myoclonic 6 (OMIM:614018)	Consider deep brain stimulation [29].
GRIN2A	Focal epilepsy, with speech disorder and with or without mental retardation (OMIM:245570)	Consider treating with N-methyl-D-aspartate receptor blockers (i.e. memantine) for gain-of-function mutations (limited evidence) [30, 31].
GRIN2B	Epileptic encephalopathy, early infantile, 27 (OMIM: 616139)	Consider L-serine (limited evidence) [32]. In several patients with gain-of-functions mutations, memantine improved awareness, behavior and sleep, but did not affect seizures [33].
GRIN2D	Epileptic encephalopathy, early infantile, 46 (OMIM:617162)	Consider treating with N-methyl-D-aspartate receptor blockers (i.e. memantine) for gain-of-function mutations (limited evidence) [34].

Gene	OMIM Phenotype	Management
KCNQ2	Epileptic encephalopathy, early infantile, 7 (OMIM:613720); Seizures, benign neonatal, 1 (OMIM:121200)	Consider ezogabine for loss-of-function variants [35]. Consider phenobarbital in pateints with 'benigh' course; sodium channel blockers (i.e. carbamazepine) are potentially effective in both 'benign' and 'Early Onset Epileptic Encephalopathy' cases [36]. Ketogenic diet (limited evidence) [17].
KCNQ3	Seizures, benign neonatal, 2 (OMIM:121201)	Carbamazepine [37].
KCNT1	Epilepsy, nocturnal frontal lobe, 5 (OMIM:615005); Epileptic encephalopathy, early infantile, 14 (OMIM:614959)	Consider quinidine for variants distal to MADP domain within RCK2; kethogenic diet; vigabatrin, especially in patients with epileptic spasms [38]. Possible use of nonnarcotic antitussive drugs (limited evidence) [39].
LGI1	Epilepsy, familial temporal lobe, 1 (OMIM:600512)	Immunotherapy with anti-epileptic drugs as add-on treatment, with carbamazepine more effective than levetiracetam [40].
MOCS1	Molybdenum cofactor deficiency A (OMIM:252150)	Consider substitution therapy with purified cyclic pyranopterin monophosphate [41].
MTOR	Focal cortical dysplasia, type II, somatic (OMIM:607341); Smith-Kingsmore syndrome (OMIM:616638)	Consider mTOR inhibitors (i.e. everolimus, limited evidence, varying effectiveness) [42, 43].
NGLY1	Congenital disorder of deglycosylation (OMIM:615273)	Theoretical support for use of endo-β-N-acetylglucosaminidase inhibitors, including proton pump inhibitors [44].
PCDH19	Epileptic encephalopathy, early infantile, 9 (OMIM:300088)	Bromide and clobazam [45]; corticosteroids [46].
PHGDH	Neu-Laxova syndrome 1 (OMIM:256520); Phosphoglycerate dehydrogenase deficiency (OMIM:601815)	Consider L-serine for reduced seizures (limited evidence) [47].
PLPBP	Epilepsy, early-onset, vitamin B6-dependent (OMIM:617290)	Pyridoxine or pyridoxal-5'-phosphate supplementation [48, 49].
PNPO	Pyridoxamine 5'-phosphate oxidase deficiency (OMIM:610090)	Pyridoxine or pyridoxal-5'-phosphate supplementation [48].
PRICKLE1, PRICKLE2	Epilepsy, progressive myoclonic 1B (OMIM:612437)	USP9X de-ubiquitinates PRICKLE, therefore USP9X inhibitor can suppress seizures (results in flies) [50].
PRRT2	Episodic kinesigenic dyskinesia 1 (OMIM:128200); Seizures, benign familial infantile, 2 (OMIM:605751); Convulsions, familial infantile, with paroxysmal choreoathetosis (OMIM:602066)	Carbamazepine is the most frequently prescribed treatment [51].

Gene	OMIM Phenotype	Management
PSAT1	Phosphoserine aminotransferase deficiency (OMIM:610992); Neu-Laxova syndrome 2 (OMIM:616038)	L-serine replacement for seizure control (limited evidence) [47].
PSPH	Phosphoserine phosphatase deficiency (OMIM:614023)	L-serine replacement [52, 53].
SCARB2	Epilepsy, progressive myoclonic 4, with or without renal failure (OMIM:254900)	Anti-myoclonic treatment in addition to anti-epileptic drugs [54].
SCN1A	Febrile seizures, familial, 3A (OMIM:604403); Migraine, familial hemiplegic, 3 (OMIM:609634); Epilepsy, generalized, with febrile seizures plus, type 2 (OMIM:604403); Epileptic encephalopathy, early infantile, 6 (Dravet syndrome) (OMIM:607208)	First line therapy for patients with Dravet Syndrome includes Valproate and Clobazam [55]. Stiripentol (in combination with with valproate and clobazam) is effective in Dravet Syndrome (randomised controlled trial) [56]. Fenfluramine is also effective in controlling seizures (randomised controlled trial) [57]. Ketogenic diet has limited evidence [17]. Cannabidiol also showed effectiveness in randomized controlled trial [58]. Serotonin (5-HT) modulators (i.e. Lorcaserin) suppressed seizures in several Dravet patients [59]. A first-generation histamine receptor (H1) antagonist, Clemizole suppressed seizure behaviour in zebrafish [59]. In vitro, Na+ current blockers decrese ubnormal ionic currants resulting from SCN1A mutations [60, 61].
SCN1B	Epileptic encephalopathy, early infantile, 52 (OMIM:617350); Atrial fibrillation, familial, 13 (OMIM:615377); Cardiac conduction defect, nonspecific (OMIM:612838); Epilepsy, generalized, with febrile seizures plus, type 1 (OMIM:604233), Autosomal dominant; Brugada syndrome 5 (OMIM:612838)	Phenytoin (limited evidence) [62].
SCN2A	Epileptic encephalopathy, early infantile, 11 (OMIM:613721); Seizures, benign familial infantile, 3 (OMIM:607745)	Sodium channel blockers recommended, Lacosamide effectiveness has limited evidence [63]. Ketogenic diet (limited evidence) [17, 64].
SCN8A	Seizures, benign familial infantile, 5 (OMIM:617080); Cognitive impairment with or without cerebellar ataxia (OMIM:614306), Autosomal dominant; Myoclonus, familial, 2 (OMIM:618364); Epileptic encephalopathy, early infantile, 13 (OMIM:614558)	High-doses of sodium channel blockers can help [65]. Zonisamide, Stiripentol, Lacosamide, rufinamide, perampanel and ketogenic diet have limited evidence [17, 55]. Sodium currant modulator GS967 is effective in suppressing seizures in animal studies [66].

Gene	OMIM Phenotype	Management
SCN9A	Small fiber neuropathy, (OMIM:133020); HSAN2D, autosomal recessive (OMIM:243000); Paroxysmal extreme pain disorder (OMIM:167400); Epilepsy, generalized, with febrile seizures plus, type 7 (OMIM:613863); Insensitivity to pain, congenital (OMIM:243000); Dravet syndrome, modifier of (OMIM:607208); Erythermalgia, primary (OMIM:133020); Febrile seizures, familial, 3B (OMIM:613863)	Oxcarbazepine, cabamazepine (limited evidence) [67, 68].
SLC12A5	Epileptic encephalopathy, early infantile, 34 (OMIM:616645); Epilepsy, idiopathic generalized, susceptibility to, 14 (OMIM:616685)	Ketogenic diet, potassium bromide (limited evidence) [69].
SLC13A5	Epileptic encephalopathy, early infantile, 25 (OMIM:615905)	Phenobarbital, valproic acid, acetazolamide [70].
SLC2A1	Dystonia 9 (OMIM:601042); GLUT1 deficiency syndrome 1, infantile onset, severe (OMIM:606777); Stomatin-deficient cryohydrocytosis with neurologic defects (OMIM:608885); GLUT1 deficiency syndrome 2, childhood onset (OMIM:612126); Epilepsy, idiopathic generalized, susceptibility to, 12 (OMIM:614847)	First line treatment is ketogenic diet [71].
SLC35A2	Congenital disorder of glycosylation, type IIm (OMIM:300896).	Oral D-galactose supplementation leads to both clinical and biochemical improvement [72].
SLC6A1	Myoclonic-atonic epilepsy (OMIM:616421)	Valproate [73].
SLC6A8	Cerebral creatine deficiency syndrome 1 (OMIM:300352)	Creatine monohydrate with l-arginine and l-glycine (limited evidence) [74, 75]. Valproate or carbamazepine for seizure control [74]. Nanoemulsion of dodecyl creatine ester showed promise in animal studies [76].
SPTAN1	Epileptic encephalopathy, early infantile, 5 (OMIM:613477)	Vigabatrin; adrenocorticotropic hormone; topiramate; clobazam; pyridoxal 5'-phosphate; levetiracetam; ketogenic diet [77].
STXBP1	Epileptic encephalopathy, early infantile, 4 (OMIM:612164)	Consider vigabatrin, valproic acid, and levetiracetam [78, 79]. Adrenocorticotropic hormone (limited evidence) [80]. Ketogenic diet (limited evidence) [17].
SUOX	Sulfite oxidase deficiency (OMIM:272300)	Low-protein diet, low cysteine and low methionine (limited evidence) [81-83].
SYNGAP1	Mental retardation, autosomal dominant 5 (OMIM:612621)	Valproate or lamotrigine are most commonly prescribed [84]. Cannabidiol (limited evidence) [85].

Gene	OMIM Phenotype	Management
TPP1	Ceroid lipofuscinosis, neuronal, 2 (CLN2, OMIM:204500); Spinocerebellar ataxia, autosomal recessive 7 (OMIM:609270)	Intraventricular administration of recombinant human tripeptidyl peptidase 1 (cerliponase alfa) for CLN2 prevents motor and language function decline, but incurs serious adverse events [86].
TSC1/TSC2	Tuberous sclerosis-1 (OMIM:191100); Focal cortical dysplasia, type II, somatic (OMIM:607341); Lymphangioleiomyomatosis, somatic (OMIM:606690)/Tuberous sclerosis-2 (OMIM:613254); Focal cortical dysplasia, type II, somatic (OMIM:607341); Lymphangioleiomyomatosis, somatic (OMIM:606690)	Vigabatrin - first line treatment for focal seizures. ACTH - second line treatment. Anti-epileptic drugs - can be started if the first-line therapy failed. Everolimus (mTOR inhibitor) and presurgical evaluation - if no response to 2 AED. Surgery is effective. In patients who fail surgeries and non0surgical patients, ketogenic diet is recommended. Vagus nerve stimulation can be used instead with ketogenic diet, or when the latter is not an option [87]. Cannabidinol shows promise in suppressing seizures [88], but increases rapamycin inhibitor levels in blood, when taken concurrently [89].

References

[1] A. Jurecka, M. Zikanova, S. Kmoch, and A. Tylki-Szymańska, "Adenylosuccinate lyase deficiency," *Journal of Inherited Metabolic Disease,* vol. 38, pp. 231-242, 2014.

[2] P. B. Mills, E. Struys, C. Jakobs, B. Plecko, P. Baxter, M. Baumgartner*, et al.*, "Mutations in antiquitin in individuals with pyridoxine-dependent seizures," *Nat Med,* vol. 12, pp. 307-9, Mar 2006.

[3] I. A. Pena, A. MacKenzie, and C. D. M. Van Karnebeek, "Current knowledge for pyridoxine-dependent epilepsy: a 2016 update," *Expert Rev Endocrinol Metab,* vol. 12, pp. 5-20, Jan 2017.

[4] B. G. Ng, E. A. Eklund, S. A. Shiryaev, Y. Y. Dong, M. A. Abbott, C. Asteggiano*, et al.*, "Predominant and novel de novo variants in 29 individuals with ALG13 deficiency: Clinical description, biomarker status, biochemical analysis, and treatment suggestions," *Journal of Inherited Metabolic Disease,* 2020.

[5] J. B. Hennermann, J.-M. Berger, U. Grieben, G. Scharer, and J. L. K. Van Hove, "Prediction of long-term outcome in glycine encephalopathy: a clinical survey," *Journal of Inherited Metabolic Disease,* vol. 35, pp. 253-261, 2011.

[6] D. A. Applegarth and J. R. Toone, "Glycine encephalopathy (nonketotic hyperglycinaemia): Review and update," *Journal of Inherited Metabolic Disease,* vol. 27, pp. 417-422, 2004.

[7] F. P. e. Vairo, B. C. Chwal, S. Perini, M. A. P. Ferreira, A. C. de Freitas Lopes, and J. A. M. Saute, "A systematic review and evidence-based guideline for diagnosis and treatment of Menkes disease," *Molecular Genetics and Metabolism,* vol. 126, pp. 6-13, 2019.

[8] M. Gasser, P. Boonsimma, W. Netbaramee, T. Wechapinan, C. Srichomthomg, C. Ittiwut*, et al.*, "ATP1A3-related epilepsy: Report of seven cases and literature-based analysis of treatment response," *Journal of Clinical Neuroscience,* vol. 72, pp. 31-38, 2020.

[9] S. Kusunoki, J. Kido, K. Momosaki, T. Sawada, T. Kashiki, S. Matsumoto*, et al.*, "Effect of Flunarizine on Alternating Hemiplegia of Childhood in a Patient with the p.E815K Mutation in ATP1A3: A Case Report," *Case Reports in Neurology,* pp. 299-306, 2020.

[10] T. Schirinzi, F. Graziola, R. Cusmai, L. Fusco, F. Nicita, M. Elia*, et al.*, "ATP1A3 -related epileptic encephalopathy responding to ketogenic diet," *Brain and Development,* vol. 40, pp. 433-438, 2018.

[11] S. I. Micó, R. D. Jiménez, E. M. Salcedo, H. A. Martínez, A. P. Mira, and C. C. Fernández, "Epilepsy in Biotinidase Deficiency After Biotin Treatment," vol. 4, pp. 75-78, 2011.

[12] H. M. Byers, C. W. Beatty, S. H. Hahn, and S. M. Gospe, "Dramatic Response After Lamotrigine in a Patient With Epileptic Encephalopathy and a De Novo CACNA1A Variant," *Pediatric Neurology,* vol. 60, pp. 79-82, 2016.

[13] L. Zhou, H. Xu, T. Wang, and Y. Wu, "A Patient With CAD Deficiency Responsive to Uridine and Literature Review," *Frontiers in Neurology,* vol. 11, 2020.

[14] Z. Lim, K. Wong, J. Downs, K. Bebbington, S. Demarest, and H. Leonard, "Vagus nerve stimulation for the treatment of refractory epilepsy in the CDKL5 Deficiency Disorder," *Epilepsy Research,* vol. 146, pp. 36-40, 2018.

[15] G. Melikishvili, N. Epitashvili, N. Tabatadze, G. Chikvinidze, O. Dulac, T. Bienvenu*, et al.*, "New insights in phenomenology and treatment of epilepsy in CDKL5 encephalopathy," *Epilepsy & Behavior,* vol. 94, pp. 308-311, 2019.

[16] Z. Lim, K. Wong, H. E. Olson, A. M. Bergin, J. Downs, and H. Leonard, "Use of the ketogenic diet to manage refractory epilepsy in CDKL5 disorder: Experience of >100 patients," *Epilepsia,* vol. 58, pp. 1415-1422, 2017.

[17] A. Ko, D. E. Jung, S. H. Kim, H.-C. Kang, J. S. Lee, S. T. Lee*, et al.*, "The Efficacy of Ketogenic Diet for Specific Genetic Mutation in Developmental and Epileptic Encephalopathy," *Frontiers in Neurology,* vol. 9, 2018.

[18] K. Lossius, A. de Saint Martin, S. Myren-Svelstad, M. Bjørnvold, G. Minken, C. Seegmuller*, et al.*, "Remarkable effect of transdermal nicotine in children with CHRNA4-related autosomal dominant sleep-related hypermotor epilepsy," *Epilepsy & Behavior,* vol. 105, p. 106944, 2020.

[19] R. P. Carson, C. Zhou, B. P. Short, and L. K. Klofas, "Prevention of premature death and seizures in a Depdc5 mouse epilepsy model through inhibition of mTORC1," *Human Molecular Genetics,* vol. 29, pp. 1365-1377, 2020.

[20] E. I. Lance, M. Kronenbuerger, J. S. Cohen, O. Furmanski, H. S. Singer, and A. Fatemi, "Successful treatment of choreo-athetotic movements in a patient with an EEF1A2 gene variant," *SAGE Open Medical Case Reports,* vol. 6, p. 2050313X1880762, 2018.

[21] I. Guella, L. Huh, M. B. McKenzie, E. B. Toyota, E. M. Bebin, M. L. Thompson*, et al.*, "De novoFGF12mutation in 2 patients with neonatal-onset epilepsy," *Neurology Genetics,* vol. 2, p. e120, 2016.

[22] F. Delmelle, B. Thöny, P. Clapuyt, N. Blau, and M.-C. Nassogne, "Neurological improvement following intravenous high-dose folinic acid for cerebral folate transporter deficiency caused by FOLR-1 mutation," *European Journal of Paediatric Neurology,* vol. 20, pp. 709-713, 2016.

[23] Y.-F. Bai, M. Chiu, E. S. Chan, P. Axerio-Cilies, J. Lu, L. Huh*, et al.*, "Pathophysiology of and therapeutic options for a GABRA1 variant linked to epileptic encephalopathy," *Molecular Brain,* vol. 12, 2019.

[24] W. Feng, S. Mei, L. Zhu, Y. Yu, W. Yang, B. Gao*, et al.*, "Effects of UGT1A6 and GABRA1 on Standardized Valproic Acid Plasma Concentrations and Treatment Effect in Children With Epilepsy in China," *Therapeutic Drug Monitoring,* vol. 38, pp. 738-743, 2016.

[25] Y. Zhang, Y. Lian, and N. Xie, "Early onset epileptic encephalopathy with a novel GABRB3 mutation treated effectively with clonazepam," *Medicine,* vol. 96, p. e9273, 2017.

[26] S. Stockler-Ipsiroglu, C. van Karnebeek, N. Longo, G. C. Korenke, S. Mercimek-Mahmutoglu, I. Marquart*, et al.*, "Guanidinoacetate methyltransferase (GAMT) deficiency: Outcomes in 48 individuals and recommendations for diagnosis, treatment and monitoring," *Molecular Genetics and Metabolism,* vol. 111, pp. 16-25, 2014.

[27] C. Brunel-Guitton, B. Casey, M. Coulter-Mackie, H. Vallance, D. Hewes, S. Stockler-Ipsiroglu*, et al.*, "Late-onset nonketotic hyperglycinemia caused by a novel homozygous missense mutation in the GLDC gene," *Molecular Genetics and Metabolism,* vol. 103, pp. 193-196, 2011.

[28] F. R. Danti, S. Galosi, M. Romani, M. Montomoli, K. J. Carss, F. L. Raymond*, et al.*, "GNAO1 encephalopathy," *Neurology Genetics,* vol. 3, p. e143, 2017.

[29] H. Yan, E. Toyota, M. Anderson, T. J. Abel, E. Donner, S. K. Kalia*, et al.*, "A systematic review of deep brain stimulation for the treatment of drug-resistant epilepsy in childhood," *Journal of Neurosurgery: Pediatrics,* vol. 23, pp. 274-284, 2019.

[30] T. M. Pierson, H. Yuan, E. D. Marsh, K. Fuentes-Fajardo, D. R. Adams, T. Markello*, et al.*, "GRIN2A mutation and early-onset epileptic encephalopathy: personalized therapy with memantine," *Annals of Clinical and Translational Neurology,* vol. 1, pp. 190-198, 2014.

[31] A. Mir, M. Qahtani, and S. Bashir, "GRIN2A-Related Severe Epileptic Encephalopathy Treated with Memantine: An Example of Precision Medicine," *Journal of Pediatric Genetics,* vol. 09, pp. 252-257, 2019.

[32] D. Soto, M. Olivella, C. Grau, J. Armstrong, C. Alcon, X. Gasull, *et al.*, "l-Serine dietary supplementation is associated with clinical improvement of loss-of-function GRIN2B-related pediatric encephalopathy," *Science Signaling,* vol. 12, p. eaaw0936, 2019.
[33] K. Platzer, H. Yuan, H. Schutz, A. Winschel, W. Chen, C. Hu, *et al.*, "GRIN2B encephalopathy: novel findings on phenotype, variant clustering, functional consequences and treatment aspects," *J Med Genet,* vol. 54, pp. 460-470, Jul 2017.
[34] D. Li, H. Yuan, X. R. Ortiz-Gonzalez, E. D. Marsh, L. Tian, E. M. McCormick, *et al.*, "GRIN2D Recurrent De Novo Dominant Mutation Causes a Severe Epileptic Encephalopathy Treatable with NMDA Receptor Channel Blockers," *Am J Hum Genet,* vol. 99, pp. 802-816, Oct 6 2016.
[35] J. J. Millichap, K. L. Park, T. Tsuchida, B. Ben-Zeev, L. Carmant, R. Flamini, *et al.*, "KCNQ2encephalopathy," *Neurology Genetics,* vol. 2, p. e96, 2016.
[36] M. Kuersten, M. Tacke, L. Gerstl, H. Hoelz, C. v. Stülpnagel, and I. Borggraefe, "Antiepileptic therapy approaches in KCNQ2 related epilepsy: A systematic review," *European Journal of Medical Genetics,* vol. 63, p. 103628, 2020.
[37] T. T. Sands, M. Balestri, G. Bellini, S. B. Mulkey, O. Danhaive, E. H. Bakken, *et al.*, "Rapid and safe response to low-dose carbamazepine in neonatal epilepsy," *Epilepsia,* vol. 57, pp. 2019-2030, 2016.
[38] M. P. Fitzgerald, M. Fiannacca, D. M. Smith, T. S. Gertler, B. Gunning, S. Syrbe, *et al.*, "Treatment Responsiveness in KCNT1-Related Epilepsy," *Neurotherapeutics,* vol. 16, pp. 848-857, 2019.
[39] C. Takase, K. Shirai, Y. Matsumura, T. Watanabe, A. Watanabe, A. Hirasawa-Inoue, *et al.*, "KCNT1-positive epilepsy of infancy with migrating focal seizures successfully treated with nonnarcotic antitussive drugs after treatment failure with quinidine: A case report," *Brain and Development,* vol. 42, pp. 607-611, 2020.
[40] M. A. A. M. de Bruijn, A. van Sonderen, M. H. van Coevorden-Hameete, A. E. M. Bastiaansen, M. W. J. Schreurs, R. P. W. Rouhl, *et al.*, "Evaluation of seizure treatment in anti-LGI1, anti-NMDAR, and anti-GABABR encephalitis," *Neurology,* vol. 92, pp. e2185-e2196, 2019.
[41] A. Veldman, J. A. Santamaria-Araujo, S. Sollazzo, J. Pitt, R. Gianello, J. Yaplito-Lee, *et al.*, "Successful treatment of molybdenum cofactor deficiency type A with cPMP," *Pediatrics,* vol. 125, pp. e1249-54, May 2010.
[42] Q. Xu, S. Uliel-Sibony, C. Dunham, H. Sarnat, L. Flores-Sarnat, L. Brunga, *et al.*, "mTOR Inhibitors as a New Therapeutic Strategy in Treatment Resistant Epilepsy in Hemimegalencephaly: A Case Report," *J Child Neurol,* vol. 34, pp. 132-138, Mar 2019.
[43] N. Hadouiri, V. Darmency, L. Guibaud, A. Arzimanoglou, A. Sorlin, V. Carmignac, *et al.*, "Compassionate use of everolimus for refractory epilepsy in a patient with MTOR mosaic mutation," *European Journal of Medical Genetics,* vol. 63, p. 104036, 2020.
[44] Y. Bi, M. Might, H. Vankayalapati, and B. Kuberan, "Repurposing of Proton Pump Inhibitors as first identified small molecule inhibitors of endo -β- N - acetylglucosaminidase (ENGase) for the treatment of NGLY1 deficiency, a rare genetic disease," *Bioorganic & Medicinal Chemistry Letters,* vol. 27, pp. 2962-2966, 2017.
[45] J. Lotte, T. Bast, P. Borusiak, A. Coppola, J. H. Cross, P. Dimova, *et al.*, "Effectiveness of antiepileptic therapy in patients with PCDH19 mutations," *Seizure,* vol. 35, pp. 106-110, 2016.
[46] N. Higurashi, Y. Takahashi, A. Kashimada, Y. Sugawara, H. Sakuma, Y. Tomonoh, *et al.*, "Immediate suppression of seizure clusters by corticosteroids in PCDH19 female epilepsy," *Seizure,* vol. 27, pp. 1-5, 2015.
[47] A. Brassier, V. Valayannopoulos, N. Bahi-Buisson, E. Wiame, L. Hubert, N. Boddaert, *et al.*, "Two new cases of serine deficiency disorders treated with l-serine," *European Journal of Paediatric Neurology,* vol. 20, pp. 53-60, 2016.

[48] M. Mastrangelo and S. Cesario, "Update on the treatment of vitamin B6 dependent epilepsies," *Expert Review of Neurotherapeutics,* vol. 19, pp. 1135-1147, 2019.
[49] X. Jiao, J. Xue, P. Gong, Y. Wu, Y. Zhang, Y. Jiang*, et al.*, "Clinical and genetic features in pyridoxine-dependent epilepsy: a Chinese cohort study," *Developmental Medicine & Child Neurology,* vol. 62, pp. 315-321, 2019.
[50] W. N. Frankel, L. Paemka, V. B. Mahajan, S. N. Ehaideb, J. M. Skeie, M. C. Tan*, et al.*, "Seizures Are Regulated by Ubiquitin-specific Peptidase 9 X-linked (USP9X), a De-Ubiquitinase," *PLOS Genetics,* vol. 11, p. e1005022, 2015.
[51] D. Ebrahimi-Fakhari, A. Saffari, A. Westenberger, and C. Klein, "The evolving spectrum ofPRRT2-associated paroxysmal diseases," *Brain,* vol. 138, pp. 3476-3495, 2015.
[52] M. Maugard, P.-A. Vigneron, J. P. Bolaños, and G. Bonvento, "l-Serine links metabolism with neurotransmission," *Progress in Neurobiology,* p. 101896, 2020.
[53] H. M. Byers, R. L. Bennett, E. A. Malouf, M. D. Weiss, J. Feng, C. R. Scott*, et al.*, "Novel Report of Phosphoserine Phosphatase Deficiency in an Adult with Myeloneuropathy and Limb Contractures," vol. 30, pp. 103-108, 2015.
[54] W.-T. Tian, X.-L. Liu, Y.-Q. Xu, X.-J. Huang, H.-Y. Zhou, Y. Wang*, et al.*, "Progressive myoclonus epilepsy without renal failure in a Chinese family with a novel mutation in SCARB2 gene and literature review," *Seizure,* vol. 57, pp. 80-86, 2018.
[55] E. Musto, E. Gardella, and R. S. Møller, "Recent advances in treatment of epilepsy-related sodium channelopathies," *European Journal of Paediatric Neurology,* vol. 24, pp. 123-128, 2020.
[56] C. Chiron, M. C. Marchand, A. Tran, E. Rey, P. d'Athis, J. Vincent*, et al.*, "Stiripentol in severe myoclonic epilepsy in infancy: a randomised placebo-controlled syndrome-dedicated trial," *The Lancet,* vol. 356, pp. 1638-1642, 2000.
[57] R. Nabbout, A. Mistry, S. Zuberi, N. Villeneuve, A. Gil-Nagel, R. Sanchez-Carpintero*, et al.*, "Fenfluramine for Treatment-Resistant Seizures in Patients With Dravet Syndrome Receiving Stiripentol-Inclusive Regimens," *JAMA Neurology,* vol. 77, p. 300, 2020.
[58] O. Devinsky, J. H. Cross, L. Laux, E. Marsh, I. Miller, R. Nabbout*, et al.*, "Trial of Cannabidiol for Drug-Resistant Seizures in the Dravet Syndrome," *New England Journal of Medicine,* vol. 376, pp. 2011-2020, 2017.
[59] A. Griffin, K. R. Hamling, K. Knupp, S. Hong, L. P. Lee, and S. C. Baraban, "Clemizole and modulators of serotonin signalling suppress seizures in Dravet syndrome," *Brain,* p. aww342, 2017.
[60] R. Barbieri, S. Bertelli, M. Pusch, and P. Gavazzo, "Late sodium current blocker GS967 inhibits persistent currents induced by familial hemiplegic migraine type 3 mutations of the SCN1A gene," *The Journal of Headache and Pain,* vol. 20, 2019.
[61] B. Terragni, P. Scalmani, E. Colombo, S. Franceschetti, and M. Mantegazza, "Ranolazine vs phenytoin: greater effect of ranolazine on the transient Na+ current than on the persistent Na+ current in central neurons," *Neuropharmacology,* vol. 110, pp. 223-236, 2016.
[62] L. T. Dang, S. C. Quinonez, B. R. Becka, L. L. Isom, and S. M. Joshi, "Dramatic Improvement in Seizures With Phenytoin Treatment in an Individual With Refractory Epilepsy and a SCN1B Variant," *Pediatric Neurology,* vol. 108, pp. 121-122, 2020.
[63] F.-H. Hadar, H. Eli, L. Ayelet, R. Orit, W. Nathan, L. Ita*, et al.*, "Lacosamide for SCN2A-related intractable neonatal and infantile seizures," *Epileptic Disorders,* vol. 20, pp. 440-446, 2018.
[64] D. Turkdogan, G. Thomas, and B. Demirel, "Ketogenic diet as a successful early treatment modality for SCN2A mutation," *Brain and Development,* vol. 41, pp. 389-391, 2019.
[65] R. S. Boerma, K. P. Braun, M. P. H. van de Broek, F. M. C. van Berkestijn, M. E. Swinkels, E. O. Hagebeuk*, et al.*, "Remarkable Phenytoin Sensitivity in 4 Children with

SCN8A-related Epilepsy: A Molecular Neuropharmacological Approach," *Neurotherapeutics,* vol. 13, pp. 192-197, 2015.
[66] E. M. Baker, C. H. Thompson, N. A. Hawkins, J. L. Wagnon, E. R. Wengert, M. K. Patel*, et al.*, "The novel sodium channel modulator GS-458967 (GS967) is an effective treatment in a mouse model of SCN8A encephalopathy," *Epilepsia,* vol. 59, pp. 1166-1176, 2018.
[67] S. Zhang, Z. Zhang, Y. Shen, Y. Zhu, K. Du, J. Guo*, et al.*, "SCN9A Epileptic Encephalopathy Mutations Display a Gain-of-function Phenotype and Distinct Sensitivity to Oxcarbazepine," *Neuroscience Bulletin,* vol. 36, pp. 11-24, 2019.
[68] S. Heil, M. Pringsheim, C. Betzler, J. Hoffmann, T. Grau, K. Hörtnagel*, et al.*, "PP07.15 – 2633: Familial generalized epilepsy with absences with SCN9A mutation: Therapy with carbamazepine," *European Journal of Paediatric Neurology,* vol. 19, pp. S59-S60, 2015.
[69] T. Saito, A. Ishii, K. Sugai, M. Sasaki, and S. Hirose, "A de novo missense mutation inSLC12A5found in a compound heterozygote patient with epilepsy of infancy with migrating focal seizures," *Clinical Genetics,* vol. 92, pp. 654-658, 2017.
[70] Q.-Z. Yang, E. M. Spelbrink, K. L. Nye, E. R. Hsu, and B. E. Porter, "Epilepsy and EEG Phenotype of SLC13A5 Citrate Transporter Disorder," *Child Neurology Open,* vol. 7, p. 2329048X2093136, 2020.
[71] J. Klepper, C. Akman, M. Armeno, S. Auvin, M. Cervenka, H. J. Cross*, et al.*, "Glut1 Deficiency Syndrome (Glut1DS): State of the art in 2020 and recommendations of the international Glut1DS study group," *Epilepsia Open,* vol. 5, pp. 354-365, 2020.
[72] P. Witters, S. Tahata, R. Barone, K. Õunap, R. Salvarinova, S. Grønborg*, et al.*, "Clinical and biochemical improvement with galactose supplementation in SLC35A2-CDG," *Genetics in Medicine,* vol. 22, pp. 1102-1107, 2020.
[73] A. Posar and P. Visconti, "Mild Phenotype Associated with SLC6A1 Gene Mutation: A Case Report with Literature Review," *J Pediatr Neurosci,* vol. 14, pp. 100-102, Apr-Jun 2019.
[74] A. Schulze, "Creatine deficiency syndromes," vol. 113, pp. 1837-1843, 2013.
[75] S. Mercimek-Mahmutoglu, M. B. Connolly, K. J. Poskitt, G. A. Horvath, N. Lowry, G. S. Salomons*, et al.*, "Treatment of intractable epilepsy in a female with SLC6A8 deficiency," *Molecular Genetics and Metabolism,* vol. 101, pp. 409-412, 2010.
[76] G. Ullio-Gamboa, K. C. Udobi, S. Dezard, M. K. Perna, K. N. Miles, N. Costa*, et al.*, "Dodecyl creatine ester-loaded nanoemulsion as a promising therapy for creatine transporter deficiency," *Nanomedicine,* vol. 14, pp. 1579-1593, 2019.
[77] S. Syrbe, F. L. Harms, E. Parrini, M. Montomoli, U. Mütze, K. L. Helbig*, et al.*, "Delineating SPTAN1 associated phenotypes: from isolated epilepsy to encephalopathy with progressive brain atrophy," *Brain,* vol. 140, pp. 2322-2336, 2017.
[78] D. Abramov, N. G. L. Guiberson, and J. Burré, "STXBP1 encephalopathies: Clinical spectrum, disease mechanisms, and therapeutic strategies," *Journal of Neurochemistry,* 2020.
[79] R. Dilena, P. Striano, M. Traverso, M. Viri, G. Cristofori, L. Tadini*, et al.*, "Dramatic effect of levetiracetam in early-onset epileptic encephalopathy due to STXBP1 mutation," *Brain and Development,* vol. 38, pp. 128-131, 2016.
[80] S. Liu, L. Wang, X. T. Cai, H. Zhou, D. Yu, and Z. Wang, "Therapeutic benefits of ACTH and levetiracetam in STXBP1 encephalopathy with a de novo mutation," *Medicine,* vol. 97, p. e0663, 2018.
[81] H. Claerhout, P. Witters, L. Régal, K. Jansen, M.-R. Van Hoestenberghe, J. Breckpot*, et al.*, "Isolated sulfite oxidase deficiency," *Journal of Inherited Metabolic Disease,* vol. 41, pp. 101-108, 2017.

[82] M. Boyer, M. Sowa, R. Wang, and J. Abdenur, "Isolated Sulfite Oxidase Deficiency: Response to Dietary Treatment in a Patient with Severe Neonatal Presentation," *Journal of Inborn Errors of Metabolism and Screening,* vol. 7, 2019.
[83] S. Rocha, A. C. Ferreira, A. I. Dias, J. P. Vieira, and S. Sequeira, "Sulfite oxidase deficiency – An unusual late and mild presentation," *Brain and Development,* vol. 36, pp. 176-179, 2014.
[84] D. R. M. Vlaskamp, B. J. Shaw, R. Burgess, D. Mei, M. Montomoli, H. Xie*, et al.*, "SYNGAP1 encephalopathy: A distinctive generalized developmental and epileptic encephalopathy," *Neurology,* vol. 92, pp. e96-e107, Jan 8 2019.
[85] M. Kuchenbuch, G. D'Onofrio, N. Chemaly, G. Barcia, T. Teng, and R. Nabbout, "Add-on cannabidiol significantly decreases seizures in 3 patients with SYNGAP1 developmental and epileptic encephalopathy," *Epilepsia Open,* vol. 5, pp. 496-500, 2020.
[86] A. Schulz, T. Ajayi, N. Specchio, E. de Los Reyes, P. Gissen, D. Ballon*, et al.*, "Study of Intraventricular Cerliponase Alfa for CLN2 Disease," *New England Journal of Medicine,* vol. 378, pp. 1898-1907, 2018.
[87] P. Curatolo, R. Nabbout, L. Lagae, E. Aronica, J. C. Ferreira, M. Feucht*, et al.*, "Management of epilepsy associated with tuberous sclerosis complex: Updated clinical recommendations," *European Journal of Paediatric Neurology,* vol. 22, pp. 738-748, 2018.
[88] E. Thiele, E. M. Bebin, F. Filloux, P. Kwan, R. Loftus, F. Sahebkar*, et al.*, "Long-term Safety and Efficacy of Cannabidiol (CBD) for the Treatment of Seizures in Patients with Tuberous Sclerosis Complex (TSC) in an Open-label Extension (OLE) Trial (GWPCARE6) (677)," *Neurology,* vol. 94, p. 677, 2020.
[89] D. Ebrahimi-Fakhari, K. D. Agricola, C. Tudor, D. Krueger, and D. N. Franz, "Cannabidiol Elevates Mechanistic Target of Rapamycin Inhibitor Levels in Patients With Tuberous Sclerosis Complex," *Pediatric Neurology,* vol. 105, pp. 59-61, 2020.
