# Supplementary material for: The Implementation Science for Genomic Health Translation (INSIGHT) Study in Epilepsy: Protocol for a Learning Health Care System
Source: JMIR Res Protoc. 2021 Mar 26;10(3):e25576. doi: 10.2196/25576 (PMC8088873; doi:10.2196/25576)
Supplement: Multimedia Appendix 2 [file resprot_v10i3e25576_app2.pdf]

Multimedia Appendix 2. Known Pharmacogenetic loci in epilepsy.

Gene	Management
CYP2C9	In intermediate and poor metabolizers (*2 and /or *3 allele carriers), reduce maintenance dose of phenytoin [1-3].
HLA-B	Avoid phenytoin, fosphenytoin, oxcarbazepine and carbamazepine use in HLA-B*1502 carriers [1, 2].
HLA-A	Avoid carbamazepine use in HLA-A*3101 carriers [1, 2].
Urea Cycle Disorders (ABL2, ASL, ASS1, CPS1, NAGS, OTC)	Valproic acid is contraindicated [1, 2].
POLG	Valproic acid and divalproex sodium are contraindicated in patients with hereditary neurometabolic syndromes caused by D mutations [1, 2].
SCN1A	Carriers of T allele of rs3812718 have increased risk of seizures, increased dose and resistance to carbamazepine, and increased dose of phenytoin [1, 3].
CYP2C19	CYP2C19 poor metabolizers may require brivaracetam and clobazam dose reduction [1-3].
EPHX1	Carriers of allele G of rs2234922 have increased dose and decreased metabolism of carbamazepine [1, 3]. Carriers of allele C of rs1051740 have increased metabolism and dose of carbamazepine [3].
UGT1A4	Carriers of allele T of rs2011425 have increased response and concentrations of lamotrigine [1, 3].

References

[1] M. V. Relling and T. E. Klein, "CPIC: Clinical Pharmacogenetics Implementation Consortium of the Pharmacogenomics Research Network," *Clinical Pharmacology & Therapeutics,* vol. 89, pp. 464-467, 2011.

[2] FDA. (2020, 10). *Table of Pharmacogenomic Biomarkers in Drug Labeling*. Available: https://www.fda.gov/media/124784/download

[3] M. Whirl-Carrillo, E. M. McDonagh, J. M. Hebert, L. Gong, K. Sangkuhl, C. F. Thorn*, et al.*, "Pharmacogenomics knowledge for personalized medicine," *Clin Pharmacol Ther,* vol. 92, pp. 414-7, Oct 2012.
